# Etiology and molecular pathogenesis of RA; how can we best use European initiatives to advance our knowledge?

**DOI:** 10.1186/ar3558

**Published:** 2012-02-09

**Authors:** Lars Klareskog

**Affiliations:** 1 Rheumatology Unit, Department of Medicine, Karolinska Institutet/Karolinska University Hospital, 171 76 Stockholm, Sweden

## 

Understanding etiology and molecular pathogenesis of rheumatoid arthritis is key to the development of precise prevention and curative therapy for this disease. Recent progress on how genes and environment interact in causing immune reactions that may induce arthritis in humans as well as in mice, have provided a conceptual basis for the development of new prevention and treatment strategies which need to be different for different subsets of RA. In order to bring this emerging knowledge to the level where basic and clinical academic science can collaborate with industry for rapid development of the potential new therapies, there is a need for closer collaboration between basic and clinical scientists from many centers, and for increased collaboration between industry and academia in translational medicine.

In Europe, both the EU-funded framework programs and the EU and industry funded Innovative Medicine Initiative (IMI) funder programs in rheumatology are geared to accomplishing these goals. This presentation will be concerned both with the scientific basis of these programs and with a descriptions of the challenges and potential promises that these new collaborative programs offer to rheumatology.

